# Factors associated with prenatal folic acid and iron supplementation among 21,889 pregnant women in Northern Tanzania: A cross-sectional hospital-based study

**DOI:** 10.1186/1471-2458-12-481

**Published:** 2012-06-26

**Authors:** Olukemi Ogundipe, Cathrine Hoyo, Truls Østbye, Olola Oneko, Rachael Manongi, Rolv Terje Lie, Anne Kjersti Daltveit

**Affiliations:** 1 Duke University School of Medicine, Durham, NC, USA; 2 Duke Global Health Institute, Duke University, Durham, NC, USA; 3 Department of Obstetrics and Gynecology, Kilimanjaro Christian Medical Centre, Tumaini University, Moshi, Tanzania; 4 Department of Community Health, Kilimanjaro Christian Medical College, Tumaini University, Moshi, Tanzania; 5 Department of Public Health and Primary Health Care, University of Bergen, Bergen, Norway; 6 Medical Birth Registry of Norway, Norwegian Institute of Public Health, Bergen, Norway

**Keywords:** Folic acid, Iron, Supplement, Pregnant women, Tanzania

## Abstract

**Background:**

Folate and iron deficiency during pregnancy are risk factors for anaemia, preterm delivery, and low birth weight, and may contribute to poor neonatal health and increased maternal mortality. The World Health Organization recommends supplementation of folic acid (FA) and iron for all pregnant women at risk of malnutrition to prevent anaemia. We assessed the use of prenatal folic acid and iron supplementation among women in a geographical area with a high prevalence of anaemia, in relation to socio-demographic, morbidity and health services utilization factors.

**Methods:**

We analysed a cohort of 21,889 women who delivered at Kilimanjaro Christian Medical Centre (KCMC), Moshi, Tanzania, between 1999 and 2008. Logistic regression models were used to describe patterns of reported intake of prenatal FA and iron supplements.

**Results:**

Prenatal intake of FA and iron supplements was reported by 17.2% and 22.3% of pregnant women, respectively. Sixteen percent of women reported intake of both FA and iron. Factors positively associated with FA supplementation were advanced maternal age (OR = 1.17, 1.02-1.34), unknown HIV status (OR = 1.54, 1.42-1.67), a diagnosis of anaemia during pregnancy (OR = 12.03, 9.66-14.98) and indicators of lower socioeconomic status. Women were less likely to take these supplements if they reported having had a malaria episode before (OR = 0.57, 0.53-0.62) or during pregnancy (OR = 0.45, 0.41-0.51), reported having contracted other infectious diseases (OR = 0.45, 0.42-0.49), were multiparous (OR = 0.73, 0.66-0.80), had preeclampsia/eclampsia (OR = 0.48, 0.38-0.61), or other diseases (OR = 0.55, 0.44-0.69) during pregnancy. Similar patterns of association emerged when iron supplementation alone and supplementation with both iron and FA were evaluated.

**Conclusions:**

FA and iron supplementation are low among pregnant women in Northern Tanzania, in particular among women with co-morbidities before or during pregnancy*.* Attempts should be made to increase supplementation both in general and among women with pregnancy complications.

## Background

Folate deficiency at conception and in early pregnancy is associated with increased risk of neural tube defects [1,2]. In both developing and developed countries, supplementation with 400 micrograms per day (μg/d) of folic acid is recommended for all women of child-bearing age [3,4]. Whereas men generally have iron homeostasis even in settings where dietary iron is low, in women both before pregnancy due to blood loss resulting from menses and during pregnancy, the body’s capacity to upregulate iron absorption is often insufficient [5]. The use of prophylactic iron supplements among pregnant women in developed countries is controversial, as the benefits of such supplementation in the absence of anaemia have not been firmly established [6,7]. However, the current WHO guidelines recommend that all pregnant women in areas where anaemia is highly prevalent receive supplements of both iron and folic acid [4]. Both folate and iron deficiency during pregnancy are risk factors for anaemia, preterm delivery, and low birth weight, and may contribute to poor neonatal health and increased maternal mortality [4,8,9].

There is very little data on the prevalence of neural tube defects at birth in Tanzania, and the primary indication for both folic acid and iron supplementation in this population is the prevention or treatment of anaemia during pregnancy. Folic acid and iron supplementation in this population has also been linked to reduced risk of hypertension in HIV positive pregnant women and malaria in their offspring [10,11]. The most recent country wide data from Tanzania on the prevalence of anaemia in pregnancy indicates that as many as 58% of Tanzanian women are anaemic during pregnancy [12]. In 20% anaemia was severe (Hb < 8.5 g/dl) enough to require a referral [13]. A study from rural Tanzania showed that about 60% of pregnant women had depleted iron stores and that anemia in pregnancy was associated with markers of infection and other nutritional deficiencies [14].

Recommendations for iron and folic acid in pregnancy in Tanzania follow the World Health Organization (WHO) recommendations of 60 milligrams (mg) of iron in addition to folic acid daily for all pregnant women at risk of malnutrition to prevent anaemia [4]. The strong evidence supporting folic acid supplementation during pregnancy, emphasized in the WHO iron guidelines, have led to wide prescription of folic acid and iron pre-conceptionally and prenatally in developing countries [15]. However, adherence to supplementation varies greatly and is influenced by socio-demographic and health factors [16-21]. In this study, we explore how folic acid and iron supplementation during pregnancy in Northern Tanzania corresponds with the recommendations, and to assess the extent to which socio-demographic indicators, health before or during pregnancy, or health care factors are associated with such supplementation.

## Methods

### Study population

In this hospital based cross-sectional observational study we utilize data from the Kilimanjaro Christian Medical College (KCMC). The hospital is one of four zonal referral hospitals in Tanzania. It serves the local community and receives medical referrals from the larger Kilimanjaro region (population ~10 million). Most patients are indigenous Chagga and Pare, although over 60 other ethnic groups including Sambaa, Sukuma, Rangi and Maasai are also represented [22].

Births occurring at KCMC after 1999 have been electronically recorded in a birth registry housed at KCMC. Methods for recruiting participants for this registry have been described in detail elsewhere [22]. Briefly, the birth registry is part of a collaboration between the University of Bergen, Norway and the KCMC, Tumaini University in Moshi Urban District of the Kilimanjaro region in Northern Tanzania. Women admitted for delivery are asked to bring a copy of their antenatal care (ANC) card, a medical record of visits during pregnancy. Within 24 hours of delivery and after maternal verbal consent, a registry midwife conducts a structured interview with the mother and abstracts information on antenatal visits for the medical record. The registry combines ANC data and pregnancy outcomes data.

From February 1st 1999 to December 31st 2008, 26,152 births were included in the birth registry. We excluded referrals due to pregnancy or labour complications (n = 3,582) as they generally do not receive antenatal care at KCMC. To ensure that each pregnancy was counted only once since our unit of analysis was the pregnancy and not the birth, we excluded second twins and second and third triplets (n = 851), leaving 21,889 pregnancies in the analyses sample. Each pregnancy was included as a separate observation for women who delivered more than once during the 10 year period. The overall registry study was approved by the KCMC Ethics Committee and the Ministry of Health in Tanzania. The protocol for the current analyses was approved by the Duke University Medical Center Institutional Review Board.

### Description of variables

#### Folic acid and iron supplementation

Information on folic acid and iron was retrieved from the antenatal record where there are separate checkboxes for folic acid and iron supplementation. Information was further obtained by interview with responses “Yes” or “No” to the questions “Did you take any drugs during this pregnancy?” The questions allowed women to report intake of these supplements whether they were prescribed and issued at the clinic, which is a common practice, or purchased over the counter from somewhere else than the KCMC pharmacy. Neither supplementation dose nor timing of when supplementation began, were collected. Supplementation of folic acid and iron were treated as binary outcomes in all analyses.

#### Socio-demographic correlates of supplementation

Socio-demographic correlates of supplement use included marital status (married, single, divorced, widowed); maternal age in years (<18, 18–25, 26–35,>35); parity (nulliparous, parous); education level of the woman and her partner (none, primary (1–7 years), secondary (8–11 years), tertiary (12 or more years)); occupation in categories of professional denoting requiring training (teachers, doctors, nurses, midwives and other occupations normally requiring education beyond secondary school level), housewife (housework, care of offspring), farmer (plant or animal husbandry in family farm in addition to housework and caring for offspring), service (typically, ward attendant in hospital, cleaner, bus driver, shopkeeper and messenger), business (denotes shop owners or women who conduct other trades, with measurable incomes), and other (including students, hairdressers, or house-girls) and tribe of participant and her partner categorized as Chagga, Pare and other. To avoid many and small categories in the multiple regression analysis, we grouped some of the categories for maternal age, parental education and parental occupation.

#### Health indicators and healthcare utilization

Health indicators based on structured interview with the mother and with supplementary information from the antenatal record and standard protocols were episodes of malaria, anaemia, or infections. Infections were defined as any infectious condition other than schistosomiasis, tuberculosis, or malaria. “Other diseases before pregnancy” included diabetes, heart disease, epilepsy, liver disease, sickle cell, TB, and lung disease. “Other diseases during pregnancy” included gestational diabetes, tuberculosis, diabetes, hypertension, epilepsy, bleeding, jaundice, schistosomiasis, heart disease and lung disease. Health care utilization variables included number of prenatal care visits categorized into few or no visits (0–1), 2–3 visits, 4–8 visits (recommended number of visits in uncomplicated pregnancies [12], and 9–15 visits (more frequent than once a month), as well as knowledge of HIV infection status. The diagnosis of anaemia was based on haemoglobin levels below 8.5 grams per decilitre at any time during pregnancy.

### Statistical analyses

We estimated the proportion of women who ever used folic acid (yes/no), iron (yes/no) or both supplements (yes/no) during the prenatal period (percentages and 95% confidence intervals). Contingency tables were employed to estimate crude odds ratios (ORs) with 95% confidence intervals for the association between each of the three dichotomous outcome variables, and socio-demographic, health services utilization and pre-pregnancy and pregnancy related morbidity. All factors associated with iron or folic acid supplementation in bivariate analyses (defined as p value ≤ 0.20) were included in a multiple logistic regression model to identify those independently associated with supplementation. Due to missing values, the number of observations in the multiple regression analysis was 21,027 (862 observations had missing values in one or more variables). All analytical tests were carried out using STATA/IC 10.1™ and SPSS 18.0 for Windows (SPSS Inc., Chicago, IL).

## Results

Folic acid supplementation was reported by 3,758 women (17.2%, 95% CI = 16.2-18.2%); iron supplementation by 4,887 women (22.3%, 95%CI = 21.7-22.9%) and 3,508 women (16.1%, 95%CI = 15.5-16.5%) reported taking both supplements. The distributions of socio-demographics, morbidity and health care utilization by supplement use are summarized in Tables 1 and 2. Most women were Chagga (57.9%) as were their partners (52.1%), and most were delivering their second or later child (60.8%), were married (89.5%), and had only primary education (61.0%). The most common occupation among the women was housewife (25.6%), while business (32.2%) was the most common occupation among their partners. Nearly all women (98.9%) had received some antenatal care during pregnancy but 26% had less than the recommended minimum of four antenatal visits per pregnancy. More than half (52.7%) did not knew their HIV status, this proportion declined from around 90% percent in 2000 to six percent in 2008 when HIV testing and anti-retroviral therapy became part of clinical practice at KCMC [23]. The most common medical conditions during pregnancy were malaria (20.4%) and infections (40.8%).

**Table 1:** Socio-demographic characteristics of pregnant women receiving folic acid and iron

**N**^**a**^**(%)**	**% Receiving folic acid (n = 3,758)**	**Chi square p-value**	**% Receiving Iron (n = 4,887)**	**Chi-square p-value**	**% Receiving iron + folate (n = 3,508)**	**Chi square p-value**
**Woman's marital status**						
Married 19,598 (89.5)	16.8	***	21.9	***	15.7	***
Not Married 2,168 (9.9)	21.0		27.0		19.6	
**Woman's age**						
26-35 10,657 (48.7)	15.5		20.0		14.1	
Under 18 475 (2.2)	22.1		26.1		21.1	
18–25 8,493 (38.8)	19.3		25.4		18.3	
Over 35 2,169 (9.9)	16.3	***	20.7	***	15.5	***
**Woman's tribe**						
Chagga 12,679 (57.9)	16.4	***	21.4	***	15.3	***
Pare 2,543 (11.6)	20.0		25.0		18.9	
Other 6,611 (30.2)	17.7		23.2		16.4	
**Woman’s highest educational level**						
None 355 (1.6)	21.7	*	27.3		21.1	
Primary (1–7) 13,355 (61.0)	17.6		23.3		16.9	
Secondary (8–11) 1,112 (5.1)	16.1		21.6		15.2	
Higher (12+) 6,999 (32.0)	16.2		20.4		14.3	
**Woman's occupation**						
Professional 3,487 (15.9)	15.9	***	19.6	***	13.4	***
Housewife 5,613 (25.6)	21.3		26.2		20.3	
Farmer 3,947 (18.0)	15.3		22.1		14.7	
Service 1,605 (7.3)	15.3		20.3		14.3	
Business 5,033 (23.0)	15.7		20.0		15.0	
Other 2,026 (9.3)	16.7		24.4		15.5	
**Partner's tribe**						
Chagga 11,408 (52.1)	16.2	***	21.5	***	15.1	***
Pare 2,505 (11.4)	19.0		24.6		17.8	
Other 7,774 (35.2)	18.3		23.2		17.0	
**Partner’s highest educational level**						
None 125 (0.6)	24.8	**	32.0		24.0	
Primary (1–7) 10,812 (49.4)	17.9		23.6		17.1	
Secondary (8–11) 836 (3.8)	17.1		22.1		16.0	
Higher (12+) 10,011 (45.7)	16.3		20.9		14.8	
**Partner's occupation**						
Professional 4,641 (21.2)	15.6	***	19.4	***	13.5	***
Farmer 2,036 (9.3)	20.3		26.2		19.4	
Business 7,047 (32.2)	16.1		21.3		15.0	
Skilled worker 2,894 (13.2)	10.6		19.3		10.2	
Other 707 (3.2)	18.8		27.2		17.3	
Service 4,464 (20.4)	23.1		26.5		22.3	

Northern Tanzania 1999-2008.

* p < 0.05.

** p < 0.01.

*** p < 0.001.

^a^The numbers do not add to the total due to missing values.

**Table 2:** Morbidity and health services usage data and percentages of pregnant women receiving folic acid and iron

**N**^**a**^**(%)**	**% Receiving folic acid (n = 3,758)**	**Chi square p-value**	**% Receiving Iron (n = 4,887)**	**Chi square p-value**	**% Receiving iron + folate (n = 3,508)**	**Chi square p-value**
**Before pregnancy**						
**Malaria**						
Y 12,605 (57.6)	12.8	***	20.4	***	11.7	***
N 9,284 (42.4)	23.1		25.0		21.9	
**Other diseases before pregnancy**						
Y 3,795 (17.3)	10.0	***	19.6	***	8.7	***
N 18,094 (82.7)	18.7		22.9		17.6	
**HIV**						
Y 795 (3.8)	18.0	***	26.4	**	16.6	***
N 11,059 (43.5)	14.8		21.5		13.4	
Status unknown 10,035 (52.7)	19.7		22.9		18.9	
**During pregnancy**						
**Parity**						
First child 8,595 (39.3)	20.3	***	26.5	***	18.8	***
Not first child 13,294 (60.8)	15.1		19.6		14.2	
**Antenatal care during pregnancy**						
Y 21,658 (98.9)	17.2	***	22.4	***	11.9	***
N 160 (0.7)	12.5		15.6		16.1	
**Number of antenatal care visits**						
“0-1” 502 (2.4)	14.5	*	18.5		13.1	*
“2-3” 5017 (23.6)	15.9		14.9		14.9	
“4-8” 14387 (67.6)	17.6		16.4		16.4	
“9-15” 1392 (6.5)	18.0		20.5		16.6	
**Preeclampsia/Eclampsia**						
Y 8881 (4.0)	10.2	***	12.5		8.5	***
N 21,008 (96.0)	17.5		22.7		16.3	
**Anaemia**						
Y 471 (2.2)	70.1	***	74.9	***	65.8	***
N 21,418 (97.9)	16.0		21.2		14.9	
**Malaria**						
Y 4,465 (20.4)	11.1	***	14.3		10.2	***
N 17,424 (79.6)	18.7		24.4		17.5	
**Infections**						
Y 8,924 (40.8)	12.2	***	15.6		10.7	***
N 12,965 (59.2)	20.6		27.0		19.7	
**Other diseases during pregnancy**						
Y 1,074 (4.9)	11.0	***	13.7		9.0	***
N 20,815 (95.1)	17.5		22.8		16.4	

Northern Tanzania 1999-2008.

* p < 0.05.

** p < 0.01.

*** p < 0.001.

^a^The numbers do not add to the total due to missing values.

In the multiple regression analysis with all presented variables included in the model (Tables 3), higher odds of folic acid supplement use were seen in women aged 35 years or older (OR = 1.17, 1.02-1.34), women from the Pare tribe (OR = 1.16, 1.02-1.32), and housewives (OR = 1.16, 1.01-1.34). Lower odds of folic acid supplementations were seen in women expecting their second or later child (OR = 0.73, 0.66-0.80) and those with few antenatal visits (OR = 0.57, 0.42-0.77 for 0–1 visits and OR = 0.72, 0.60-0.85 for 2–3 visits). With respect to maternal health, higher odds of folic acid supplement use were seen in women who did not know their HIV status (OR = 1.54, 1.42-1.67), and women who had anaemia during pregnancy (OR = 12.03, 9.66-15.0), whereas other medical conditions before or during pregnancy were associated with lower odds of folic acid supplement use; malaria before pregnancy (OR = 0.57, 0.53-0.62), malaria during pregnancy (OR = 0.45, 0.41-0.51), infectious diseases (OR = 0.45, 0.42-0.49), preeclampsia/eclampsia (OR = 0.48, 0.38-0.61), or other diseases during pregnancy (OR = 0.55, 0.44-0.69). Similar patterns of associations were seen for iron and iron/folic acid supplementation, results for these outcomes are presented in the tables. There was no trend in supplementation with folic acid or iron according to period of birth (1999–2004 vs. 2005–2008), and inclusion of year of birth in the multiple regression analysis did not change the results.

**Table 3:** Associations of predictors of antenatal folic acid and iron supplementation in Northern Tanzania 1999–2008 from the multiple regression analysis

		% Receiving folic acid (n = 3,758)a		% Receiving Iron (n = 4,887)a		% Receiving iron + folate (n = 3,508)a
		Adjusted^b^ OR (95% CI)		Adjusted^b^ OR (95% CI)		Adjusted^b^ OR (95% CI)
**Woman's marital status**						
Married	1		1		1	
Not Married		1.14(1.01–1.28)*		1.14 (0.99–1.29)		1.14 (1.00–1.29)
**Woman's age**						
26-35	1		1		1	
Under 26		1.02 (0.93-1.12)		1.05 (0.96–1.16)		1.05 (0.96-1.16)
Over 35		1.17 (1.02-1.34)*		1.25 (1.08–1.43)*		1.25 (1.08-1.43)**
**Woman's tribe**						
Chagga	1		1		1	
Pare		1.16 (1.02-1.32)*		1.16 (1.01-1.32)*		1.16 (1.01-1.32)*
Other		1.00 (0.91-1.10)		0.99 (0.89-1.09)		0.99 (0.89-1.09)
**Woman’s highest education level**						
None		1.29 (0.96-1.74)		1.34 (0.99-1.81)		1.34 (0.99-1.81)
Primary (1–7)		1.02 (0.92-1.13)		1.05 (0.95-1.16)		1.05 (0.95-1.16)
Secondary (8–11)/Higher (12+)	1		1		1	
**Woman's occupation**						
Professional	1		1		1	
Housewife		1.16 (1.01-1.34)*		1.28 (1.10-1.49)**		1.28 (1.10-1.49)**
Other		0.88 (0.77-1.01)		0.96 (0.94-1.14)		0.96 (0.84-1.11)
**Partner's tribe**						
Chagga	1		1		1	
Pare		1.13 (0.99-1.29)		1.13 (0.99-1.30)		1.13 (0.99-1.30)
Other		1.04 (0.95-1.14)		1.03 (0.94-1.14)		1.03 (0.94-1.14)
**Partner’s highest educational level**						
None		1.29 (0.80-2.08)		1.27 (0.78-2.07)		1.27 (0.78-2.07)
Primary (1–7)		1.09 (0.99-1.19)		1.08 (0.98-1.18)		1.08 (0.98-1.18)
Secondary (8–11)/Higher (12+)	1		1		1	
**Partner's occupation**						
Professional	1		1		1	
Skilled worker		0.67 (0.56-0.79)***		0.72 (0.61-0.86)		0.72 (0.61-0.86)***
Other		1.13 (1.01-1.26)*		1.20 (1.06-1.35)*		1.20 (1.06-1.35)**
**Before Pregnancy**						
Y		0.57 (0.53-0.62)***		0.55 (0.51-0.60)***		0.55 (0.50-0.60)***
N	1		1		1	
**Other diseases before pregnancy**^**c**^						
Y		0.62 (0.55-0.70)***		0.58 (0.51-0.66)***		0.58 (0.51-0.66)***
N	1		1		1	
**Maternal HIV**						
Y		1.30 (1.06-1.59)*		1.31 (1.06-1.62)*		1.31 (1.06-1.62)*
N	1		1		1	
Status unknown		1.54 (1.42-1.67)***		1.63 (1.50-1.76)***		1.63 (1.50-1.76)***
**Parity**						
First Child	1		1		1	
Not first child		0.73 (0.66-0.80)***		0.75 (0.68-0.82)***		0.75 (0.68-0.82)***
**Number of antenatal care visits**						
“0-1”		0.57 (0.42-0.77)***		0.60 (0.45-0.70)***		0.60 (0.45-0.79)**
"2-3"		0.72 (0.60-0.85)***		0.80 (0.72-0.88)***		0.80 (0.72-0.88)**
“4-8”		0.88 (0.76-1.03)		1.13 (0.97-1.33)		1.13 (0.97-1.33)
"9-15"	1		1		1	
**Preeclampsia & Eclampsia**						
Y		0.48 (0.38-0.61)***		0.42 (0.33-0.54)***		0.42 (0.33-0.54)***
N	1		1		1	
**Anemia**						
Y		12.03 (9.66-14.98)***		10.81 (8.72-13.39)***		10.81 (8.72-13.39)***
N	1		1		1	
**Malaria**						
Y		0.45 (0.41-0.51)***		0.44 (0.40-0.50)***		0.44 (0.40-0.50)***
N	1		1		1	
**Infections**						
Y		0.45 (0.42-0.49)***		0.41 (0.37-0.44)***		0.41 (0.37-0.44)***
N	1		1		1	
**Other diseases during pregnancy**^**c**^						
Y		0.55 (0.44-0.69)***		0.46 (0.36-0.59)***		0.46 (0.36-0.59)***
N	1		1		1	

^a^ Number included in the multiple regression model is 21,027 (862 observations had one or more missing values).

^b^ All variables are in the adjusted model.

^c^ “Other diseases before pregnancy” included diabetes, heart disease, epilepsy, liver disease, sickle cell, TB, and lung disease. “Other diseases during pregnancy” included gestational diabetes, tuberculosis, diabetes, hypertension, epilepsy, bleeding, jaundice, schistosomiasis, heart disease and lung disease.

* p ≤ 0.05.

** p ≤ 0.01.

*** p ≤ 0.001.

## Discussion

Our key finding was that despite the WHO recommendations and the high occurrence of adverse health conditions and birth outcomes, only one fifth of the pregnant women visiting KCMC report using iron or folic acid supplements during pregnancy. Remarkably, women with morbidity were less likely to be prescribed or use folic acid and iron supplements. The proportion of women reporting supplementation with folate and/or iron is lower than what has been found in developed countries. Countries such as the USA, Norway, and the Netherlands report folic acid supplementation among pregnant women at 43%, 35%, and 72.4%, respectively [2,4,9]. Typically, iron supplementation is higher than folic acid supplementation. In the USA and the Netherlands iron supplementation among pregnant women is 72% and 77%, respectively [18,19]. In developing countries, iron supplementation during pregnancy is as high as 70% in the Philippines and 69% in Senegal [20,21].

Our finding of decreased supplementation among women with co-morbidities (other than anaemia), also after accounting for number of antenatal visits in the adjusted analyses, contrast with other studies where women with co-morbidities are more likely to use folic acid and iron supplements during pregnancy [16-18]. However, these other studies were conducted in developed countries where diagnosis is almost always tied to treatment and where women who are recognized as having co-morbidities during pregnancy tend to receive more intensive antenatal care. This may not be the case in Northern Tanzania where the presence of chronic and infectious diseases could be an indicator of poor access to health care, a factor which also contributes to lower utilization of antenatal care services and thus use of folic acid and iron supplements. This explanation is supported by our findings that women who have more ANC visits have significantly greater odds of using folic acid and both folic acid and iron supplements. Similar results have been found in previous studies [5,20].

The WHO estimates that, globally, 41% of women suffer from anemia as result of iron deficiency resulting from low supplementation [24]. Provision of folic acid and iron supplements, in addition to a standard weekly dose of prophylactic chloroquine for malaria prophylaxis, dramatically reduced the prevalence of anaemia during pregnancy in a prospective intervention study of 1045 women in two antenatal clinics in Dar Es Salaam [13]. However, factors associated with intake of these supplements are unclear, and may be setting-specific. In a study from Senegal, iron supplementation adherence was increased in women who were offered free access to supplements compared to women who only received a prescription (86% vs. 49%). Counselling on the health benefits of supplementation significantly increased compliance among the Senegalese [21]. In our study sample, the proportion receiving supplementation was low despite a very high rate of attendance to antenatal clinics – these clinic visits presumably included counselling. Because folic acid and iron are prescribed and dispensed with a very low co-payment (US$ 0.25 per monthly dose), the low use of supplementation, may be a result of a lack of knowledge about the benefits of supplementation rather than non-availability. Interventions involving educating health care providers and ensuring availability of folic acid and iron have been shown to improve intake, as evidenced by a precipitous decrease in prevalence of anemia during the course of pregnancy in a population where provider-education was given [13].

Notably, primiparous women with anaemia during pregnancy were also found to have significantly greater odds of using any supplements than multiparous women. This is a consistent finding in studies that have considered parity as a predictive factor for folic acid and iron supplementation [7,19-21,25,26]. The common explanation for finding is that multiparous women may have a more casual attitude to supplementation especially if they have had uncomplicated pregnancies in the past.

The definition of anaemia (haemoglobin less than 8.5 g/dl) in the birth registry corresponds with national criteria which are applied at antenatal clinics for referral and further investigation. However, since prevalence estimates for anaemia usually include all individuals with haemoglobin less than 11 g/dl, which includes less severe anaemia, it is difficult to compare the prevalence seen in our study sample to the prevalence for the whole country [12]. In a well designed study of 2235 pregnant women in the Dar Es Salaam region, 60% were anaemic and 2.2% severely anaemic at the first booking [13]. An earlier study investigating haemoglobin concentrations in pregnant women in the Moshi area also showed a high prevalence of anaemia [27]. The WHO bases its recommendations for folic acid and iron supplementation mostly on data from women with anaemia defined as haemoglobin less than 11 g/dl, and the presence of anaemia, based solely on haemoglobin level, is the main indication for folic acid and iron supplementation [4]. Although hemoglobin is measured at antenatal visits, supplementation is mainly based on clinical signs of anaemia and not haemoglobin level specifically (personal communication). Increasing supplementation in this region will require antenatal care clinics at KCMC and elsewhere to reconsider their definition of anaemia to make it consistent with the WHO definition and use these criteria for prescribing and dispensing iron and folic acid supplements.

While it is clear that women with HIV will especially benefit from folic acid and iron supplementation [25], our study showed that women with unknown HIV status were more likely to use both iron and folic acid supplements. For women in Northern Tanzania, and perhaps other developing countries, special attention needs to be paid to providing supplements to women with chronic and infectious diseases prior to and during pregnancy. Only in the case of malaria prophylaxis with sulfadoxine-pyrimethamine should special consideration be given in the administration of folic acid as it reduces the efficacy of sulfadoxine as an antimalarial [26].

A major strength of this study is the large sample size. Another strength is the method of data collection through a comprehensive birth registry. The birth registry data were carefully collected and comprise information obtained from questionnaire based interviews done by designated midwives and women’s medical records. The information obtained in the questionnaires was cross checked against patient records.

A limitation of this study is that the coverage of births in the KCMC region in not complete. It is estimated that 29% of births in the KCMC catchment region occur at home [12]. KCMC is not primarily a referral hospital but also serves as a local hospital for the area, and the ethnic and socio-demographic composition of the women who deliver at KCMC is diverse. However, because of its’ status as a referral hospital, women who deliver at KCMC may represent a somewhat more privileged group than those who deliver at home or at local hospitals in the area. In order to obtain a more population based sample we excluded women who were referred for delivery at KCMC due to complications. A second limitation is the fact that health conditions were based on self-reports with supplementary information from medical records. Some of the medical diagnoses were not established by diagnostic tests and may thus be over or underestimated in this sample. A third limitation of this secondary data analysis is the availability of only dichotomous data for characterizing outcomes, as timing and frequency of use of folic acid or iron supplements was not available.

## Conclusions

The use of folic acid and iron supplementation for pregnant women in Northern Tanzania is low and does not meet WHO recommendations for the treatment of anaemia during pregnancy, despite the high number of antenatal care visits per participant. Socio-demographic factors including maternal age, occupation and partners’ occupation, maternal characteristics including parity and co-morbidities, and healthcare factors such as HIV testing, few ANC visits, infections and chronic diseases are all significantly associated with supplementation. A systematic audit of the screening and supplementation system for pregnant women at KCMC and in the region may reveal reasons for this low use. Following up our findings, further studies should consider the effects of involving the women’s partners, and developing interventions especially targeting women at increased risk in clinic. Education of health care providers, routine counselling as part of the antenatal care, follow up of adherence among women at risk, and routines to ensure supplies of supplements at distribution points, represent potential actions to improve micronutrient status of pregnant women in Tanzania.

## Competing interests

The authors declare that they have no competing interests. Statistical analysis was unfunded for this study and there were no financial incentives attached to completion of this study.

## Authors' contributions

OO: Analysis and interpretation of data, manuscript drafting. CH, TØ, AKD: Conception and design, interpretation of data, manuscript review. OO, RM, RTL: Acquisition of data, manuscript review. All authors have approved the final version.

## Pre-publication history

The pre-publication history for this paper can be accessed here:

http://www.biomedcentral.com/1471-2458/12/481/prepub
